# XBP1-Mediated BiP/GRP78 Upregulation Copes with Oxidative Stress in Mosquito Cells during Dengue 2 Virus Infection

**DOI:** 10.1155/2017/3519158

**Published:** 2017-10-01

**Authors:** Tien-Huang Chen, Yi-Hsuan Chiang, Jiun-Nan Hou, Chih-Chieh Cheng, Eny Sofiyatun, Cheng-Hsun Chiu, Wei-June Chen

**Affiliations:** ^1^Department of Public Health and Parasitology, College of Medicine, Chang Gung University, Kwei-San, Tao-Yuan, Taiwan; ^2^Graduate Institute of Biomedical Sciences, Chang Gung University, Kwei-San, Tao-Yuan, Taiwan; ^3^Environmental Health Department, Banjarnegara Polytechnic, Central Java, Indonesia; ^4^Molecular Infectious Disease Research Center, Chang Gung Memorial Hospital, Kwei-San, Tao-Yuan, Taiwan; ^5^Division of Pediatric Infectious Diseases, Department of Pediatrics, Chang Gung Children's Hospital, Chang Gung University College of Medicine, Kwei-San, Tao-Yuan, Taiwan

## Abstract

Dengue viruses (DENVs) cause dengue fever which is an important mosquito-borne disease in tropical areas. Generally, DENV does not cause cellular damage in mosquito cells. However, alterations in cytosolic calcium ions ([Ca^2+^]cyt) and the mitochondrial membrane potential (MMP), as well as accumulated reactive oxygen species (ROS), including superoxide anions (O_2_^∙−^) and hydrogen peroxide (H_2_O_2_), can be detected in C6/36 cells with DENV2 infection. Evident upregulation of BiP/GRP78 also appeared at 24 h postinfection in DENV2-infected C6/36 cells. As expression of BiP/GRP78 mRNA was reduced when the transcription factor X-box-binding protein-1 (XBP1) was knocked down in C6/36 cells, it demonstrated that BiP/GRP78 is the target gene regulated by the XBP1 signal pathway. We further demonstrated that the expression and splicing activity of XBP1 were upregulated in parallel with DENV2 infection in C6/36 cells. In C6/36 cells with BiP/GRP78 overexpression, oxidative stress indicators including [Ca^2+^]cyt, MMP, O_2_^∙−^, and H_2_O_2_ were all pushed back to normal. Taken together, DENV2 activates XBP1 at earlier stage of infection, followed by upregulating BiP/GRP78 in mosquito cells. This regulatory pathway contributes a cascade in relation to oxidative stress alleviation. The finding provides insights into elucidating how mosquitoes can healthily serve as a vector of arboviruses in nature.

## 1. Introduction

The dengue virus (DENV) consists of four serotypes that serve as etiological agents of dengue fever, which also presents severe forms of the disease including dengue hemorrhagic fever (DHF) and dengue shock syndrome (DSS) on certain occasions [1]. DENV is taxonomically classified as a member of the family Flaviviridae, the genome of which is composed of a positive-sense single-stranded RNA of ~11 kilobases (kb) in length [2]. Generally, flaviviral RNAs possess a 7-methylguanosine (m7G) cap at the 5′-end and are nonpolyadenylated at their 3′-end [2]. DENV is transmitted between humans in nature by* Aedes* mosquitoes, principally* Aedes aegypti* [3]. In turn, the DENV can alternately infect and propagate in mosquito and human cells to maintain its natural replication cycle [4]. Once a host cell is infected, viral genomic RNA is released and directly translated into a single polyprotein which is subsequently cleaved into three structural proteins and seven nonstructural proteins in the order of C-prM-E-NS1-NS2A-NS2B-NS3-NS4A-NS4B-NS5 within a membranous structure related to the endoplasmic reticulum (ER) [5]. Protein synthesis of flaviviruses in host cells usually induces hypertrophy of ER membranes [6] and thus overwhelms the ER folding capacity [7].

The ER is a site for cellular calcium storage, lipid biosynthesis, membrane biogenesis, and xenobiotic detoxification [8] and is also where proteins are folded and assembled before entering secretory pathways in eukaryotic cells [9, 10]. Stimuli that disrupt the functions of the ER due to the accumulation of misfolded and unfolded proteins in the ER lumen usually lead to the formation of ER stress which activates a signaling network called the unfolded protein response (UPR) [11]. The UPR is a relatively sophisticated signaling system, generally involving the folding and maturation of newly synthesized peptides across the ER membrane [12]. However, the UPR leads to apoptosis if the stress persists due to an inability to mitigate it within a certain time frame [13]. In fact, most mammalian cells become apoptotic in response to DENV infection and its induced ER stress [4]. It is believed that DENV-induced cell death is relevant to the pathogenesis of dengue disease in humans [14]. In contrast, DENV-infected mosquito cells mostly survive the infection, although some cytopathic effects may be shown in a small proportion of virus-infected cells [15, 16]. This indicates that the fate of an infected cell is highly dependent on its origin. In spite of this, DENV2-induced oxidative stress was shown to occur in mosquito cells infected by DENV2 [17]. However, it is usually mitigated by upregulated antioxidant defenses and/or antiapoptotic effects in response to the infection [17, 18].

Generally, ER stress induces the UPR which favors cell survival through its primary role of increasing the capacity to correctly fold proteins and effectively export unfolded or misfolded proteins to the cytosol for subsequent degradation [13]. It provides a mechanism for the quality and quantity control of synthesized viral proteins, leading to lower oxidative stress and higher survival possibilities in infected host cells. The UPR in mammalian cells is known to activate signals that are then transmitted from the ER to the cytoplasm and nucleus, resulting in expressions of target genes, mostly via three signaling pathways: PKR-like ER kinase (PERK), activating transcription factor 6 (ATF6), and inositol-requiring transmembrane protein kinase/endonuclease 1 (IRE1) [19]. PKR represents the double-stranded RNA- (dsRNA-) activated protein kinase. All the three ER-transmembrane proteins are physiologically bound to ER-resident BiP [19]. BiP is an immunoglobulin heavy-chain-binding protein, which is also known as glucose-regulated protein 78 (GRP78) and is thus referred to as BiP/GRP78 [19]. The dissociation of ER-residential BiP/GRP78 from the three transmembrane proteins during the UPR is an essential step in initiating cascades of downstream regulatory activities in response to ER stress [20]. After millions of years of coevolution with their hosts, viruses have developed relatively sophisticated strategies to hijack cellular factors and use them for sustained maintenance in nature [21]. A genome-wide transcriptomic analysis of DENV2-infected human Mo-DC (dendritic cells) demonstrated that induced oxidative stress is critical to the outcome of DENV infection in cells, in terms of both antiviral and apoptotic programs [22].

DENV infection in mosquito cells was shown to activate the translation of some genes in relation to ER stress caused by unfolded proteins [23]. We recently identified that BiP/GRP78 is upregulated in C6/36 cells with DENV2 infection [17], indicating that DENV2 infection also induces the UPR in mosquito cells despite most infected cells eventually surviving. BiP/GRP78 is known to have a correlation with X-box-binding protein-1 (XBP1), leading to downstream effects in cells [24]. Herein, we investigated the regulatory mechanism during the UPR induced by DENV2 infection, particularly the involvement of these molecules in coping with virus-induced oxidative stress in mosquito cells.

## 2. Materials and Method

### 2.1. Virus and Cell Culture

DENV type 2 (New Guinea C strain) was propagated in C6/36 cells (derived from the mosquito* Ae. albopictus*) with minimal essential medium (MEM, Invitrogen, Carlsbad, CA, USA) containing 10% fetal bovine serum (FBS), 2% nonessential amino acids, 2 g/ml Hepes (Sigma-Aldrich, St. Louis, MO, USA), 2.2 g/ml sodium bicarbonate (NaHCO_3_), and 0.4% of an antibiotic-antimycotic (Invitrogen) at 28°C in a closed system. The virus was titrated as described previously in baby hamster kidney- (BHK-) 21 cells [25] maintained in MEM containing 10% FBS, 2% nonessential amino acids, 2.2 g/ml NaHCO_3_, and 0.4% of an antibiotic-antimycotic at 37°C in an incubator under a 5% CO_2_ atmosphere. Both cell lines were kindly provided by Dr. C. L. Kao of National Taiwan University and have been maintained in our laboratory since then. Cell numbers after culture at selected time points were determined by counting viable cells with a hemocytometer after trypan blue staining. The average of three replicates is shown in the figure.

### 2.2. RNA Extraction and Detection

The methods in this part followed previously described procedures [18]. DENV2-infected or uninfected C6/36 cells (~10^7^ cells/tube) harvested from the culture were centrifuged at 3000 rpm and 4°C for 10 min and then dispensed into dishes (6 cm in diameter). The DENV2 suspension or medium (for the group with mock infection as the control) was added at a multiplicity of infection (MOI) of 1 for the group with infection. The dishes were then incubated at 28°C for 1 h with gentle agitation every 15 min. After fresh MEM was added, the dishes were then incubated at 28°C for another 24 h. RNA extraction, complementary DNA (cDNA) synthesis, and a reverse-transcription polymerase chain reaction (RT-PCR) were then performed with M-MLV reverse transcriptase (Invitrogen) following the manufacturer's instructions. The cDNA was then used to detect the virus, and genes of the cellular or viral RNAs were validated/quantified.

### 2.3. Plaque Assay for Virus Titration

A plaque assay used to determine the virus titer was implemented in baby hamster kidney-21 (BHK-21) cells, following a method described in one of our previous reports [26]. The final virus titer was calculated based on the number of plaques formed in wells of a 6-well culture plate and was expressed as plaque forming units per milliliter (PFU/ml). The results was the average of three replicates.

### 2.4. Gene Validation and Quantification of BiP/GRP78 by an RT-PCR

Reverse-transcribed cDNAs from extracted total RNA as described above were subjected to a conventional and/or a real-time quantitative RT-(q)PCR for validation and quantification of the BiP/GRP78 gene. The primer pairs, including BiP1-3F (5′-CCTACTCGTGTGTGGGAGTGTA-3′) and BiP1-3R (5′-ATGATACGCATCACGTTCAAAC) (used for the conventional RT-PCR) as well as BiP RTF (5′-ATGCCAACGGTATCCTGCAG-3′) and BiP RTR (5′-GGTTTCCGGTTCCCTTGTCT-3′) (used for the RT-qPCR), were designed from two partial sequences of BiP/GRP78 identified from previously established expressed sequence tag (EST) libraries [17]. The conventional RT-PCR followed previously described procedures [25], including activation by SuperRed PCR Master Mix (2x) composed of* Taq* DNA polymerase, the NH^4+^ buffer system, dNTPs, and MgCl_2_ (Tools, New Taipei City, Taiwan) at 95°C for 5 min, followed by 30 or 35 cycles of amplification consisting of denaturing at 95°C for 30 s, 30 s of annealing at 55°C, and extension at 72°C for 30 s. The reaction was finished after an additional 10 min for elongation. For the real-time RT-PCR, the thermal cycling protocol included 10 min of activation with DNA polymerase contained in KAPA SYBR® FAST qPCR Kit Master Mix (2x) ROX Low (KAPA Systems, Wilmington, MA, USA) and performed on an Applied Biosystem® 7500 fast real-time PCR system (Waltham, MA, USA). The thermal cycling protocol included 10 min of enzyme activation at 95°C and 40 cycles of amplification consisting of 15 s of denaturing at 95°C and 60 s of annealing-extension at 60°C. Threshold cycle (Ct) numbers were established using 7500 software vers. 2.0.5 (ABI). As an internal control, levels of 18S ribosomal RNA (rRNA) designed from the genome of* Ae. albopictus* were quantified in parallel with specific genes [25]. Results are expressed as the relative quantities of the target gene. Thus, each unit difference in relative quantities indicates a 2-fold difference in gene expression, given that, during each amplification cycle, there was doubling of gene products. Multiples of change were calculated by the formula 2^−ΔΔCt^, where ΔΔCt = ΔCt (virus-infected sample) −ΔCt (mock-infected sample) [26]. The experiment was performed in triplicate, and mean multiples of change were statistically analyzed.

### 2.5. Measurement of Cytosolic Calcium Ions in DENV2-Infected C6/36 Cells

Confluent C6/36 cells cultured in 6 cm Petri dishes were infected with DENV2 (at an MOI of 1), while those without virus inoculation were used as controls. Cells incubated for 24 and 48 h were separately harvested by adding trypsin-EDTA for 5 min followed by phosphate-buffered saline (PBS; pH 7.3) containing 10% FBS to neutralize the trypsin activity. Cells from each group harvested at different time points were mixed with the fluo-3 AM calcium indicator (Invitrogen) to a final concentration of 5 *μ*M. These were incubated in the dark for 30 min at 28°C and then centrifuged at 3000 rpm for 10 min. Pellets were washed twice with PBS after removing the supernatant and then resuspended by adding 200 *μ*l of PBS containing 10% FBS. The final cell suspension was then used to measure cytosolic calcium by flow cytometry. The experiment was performed in triplicate, and mean multiples of change were statistically analyzed.

### 2.6. Detection of the Mitochondrial Membrane Potential (MMP) in DENV2-Infected C6/36 Cells

The MMP in DENV2-infected C6/36 cells was measured using FACScan flow cytometry by a previously described method [17]. The method was implemented using a MitoCapture™ mitochondrial apoptosis detection kit, following the protocol provided by the manufacturer (BioVision, Mountain View, CA, USA). In brief, both infected and uninfected cells were pelleted and then resuspended in 1 ml of diluted solution of MitoCapture. Cells were incubated at 28°C for 15~20 min and then centrifuged at 500 ×g. Following resuspension of cells in 1 ml of prewarmed incubation buffer, the sample was quantified by flow cytometry (FACScalibur, Mountain View, CA, USA).

### 2.7. Detection of Superoxide Anions in C6/36 Cells Infected by DENV2

Superoxide anions were detected as described previously [17]. Briefly, a monolayer of C6/36 cells either with or without BiP/GRP78 overexpression was infected by the DENV2 at an MOI of 1 in a 6 cm Petri dish for 24 h. Cells were then washed with PBS and treated with trypsin-EDTA for 5 min. One milliliter of PBS containing 10% FBS was added to the cultured cell dishes and incubated with 10 *μ*M dihydroethidium (Sigma-Aldrich) at 28°C in the dark for 30 min. Cells were then harvested and analyzed by a fluorescence-activated cell sorter (FACS) with excitation at 518 nm and emission at 605 nm (FACS-Calibur flow cytometer, Becton-Dickinson, Immunofluorometry Systems, Mountain View, CA, USA). One of three replicates is shown in the figures.

### 2.8. Detection of Hydrogen Peroxide in Virus-Infected C6/36 Cells

The method followed procedures described in our previous report [18]. A monolayer of C6/36 cells (with or without transfection of a BiP/GRP78-overexpressing vector) infected with DENV2 at an MOI of 1 in a 10 cm Petri dish for various infection time periods was washed with PBS (pH 7.3) and then treated with trypsin-EDTA for 5 min. One milliliter of PBS containing 10% FBS was added to the dish, and cells were incubated with 10 *μ*M 2′,7′-dichlorofluorescein (CM-H2DCFDA) (Invitrogen) at 28°C for 30 min in the dark. Cells were then harvested and subjected to analysis by a fluorescence-activated cell sorter (BD FACScalibur) with excitation at 492~495 nm and emission at 517~527 nm.

### 2.9. Determination of the Full-Length Sequence of BiP/GRP78

Determination of the full-length sequence of BiP/GRP78 followed an approach described elsewhere [25]. Extracted total RNA was used to synthesize a fragment of BiP/GRP78 with oligo dT, and primers were derived from a fragment of the sequence from previously established EST libraries [17]. PCR products were subsequently cloned into the pGEM-T vector to sequence the 3′-end of the gene. The 5′-end of* Ae. albopictus* BiP/GRP78 was obtained using a 5′RACE system (Invitrogen) according to the manufacturer's protocol. In brief, extracted total RNA was first treated with 1 U/*μ*l DNase (Promega, Madison, WI, USA) to remove genomic DNA, from which 5′-end cDNA was generated with the gene-specific primer- (GSP-) 1, 5′-GCAGTTTCCTTCATCTTG-3′, and Superscript II™ RT (Invitrogen). dCTP was added to the tail of the 5′-end cDNA using terminal deoxynucleotidyl transferase, and then the dCTP-tailed cDNA was amplified by a PCR with GSP-2 (5′-TCCTCCGGAGCGAACACCTTATC-3′) and universal primers provided by the kit's manufacturer. The obtained sequence was used to compare sequences selected from GenBank for alignment with other mosquito species as well as production of the recombinant protein.

### 2.10. Construction and Transfection of BiP/GRP78-Expressing Vectors in C6/36 Cells

Expression vectors were based on the insect-cell-expression vector, pAC5.1-V5-His A (Invitrogen). To express enhanced green fluorescent protein (eGFP) fusion proteins, the open reading frame (ORF) of BiP/GRP78 was amplified by a PCR using primers BiP-EcoRV-F (5′-AAAGATATCATGAAGCTGCTAGTACCGTTGGCCC-3′) and BiP-NotI-HA-R (5′- AAAGCGGCCGCAAAGCGTAGTCTGGGACGTCGTATGGGTAGAGATCGTCATCTTCGCCGGCAG-3′) and then inserted into the pAC5.1-eGFP expression vector in the C terminal domain of the eGFP containing additional KDEL. To express the BiP/GRP78 protein, its ORF was amplified by a PCR using primers BiP-EcoRV-F (5′-AAAGATATCATGAAGCTGCTAGTACCGTTG-3′) and BiP-NotI-R (5′- AAAGCGGCCGCTTACAGTTCATCCTTGAGATCG-3′) and then inserted into the pAC5.1-V5-HisA vector. For transfection, C6/36 cells seeded on six-well plates were grown to 70%~80% confluence. X-tremeGene HP DNA transfection reagent (Roche Diagnostics, Mannheim, Switzerland) was mixed with expressing vectors (ratio = 3 : 1 *μ*l/*μ*g; 1 *μ*g plasmid DNA per well was used in most experiments) in basal medium (MEM, 2% nonessential amino acid, 0.0375% sodium bicarbonate, and 0.2% Hepes) at room temperature for 15 min before transfection. Subsequently, C6/36 cells were incubated with the transfection mixture for 5 h, at which time it was then replaced with complete medium.

### 2.11. Preparation of Antibodies against BiP/GRP78

To prepare the BiP/Grp78 antibodies, the pET30a/BiP/GRP78 vector with a His-tag was constructed by inserting the ORF of BiP/GRP78 (1971 bp) which was amplified by a PCR using the BiP-EcoRV-F (5′-AAAGATATCATGAAGCTGCTAGTACCGTTG-3′) and BiP-NotI-R (5′-AAAGCGGCCGCTTACAGTTCATCCTTGAGATCG-3′) primers. The fusion protein expressed by the construct was concentrated, purified, and then used to immunize rabbits, following the method implemented for other proteins in this laboratory previously [27]. Animals were bled 2 weeks after the final injection of the antigen and the efficacy of the elicited antibody in the collected antiserum was tested by Western blotting.

### 2.12. Immunofluorescence Assay (IFA)

C6/36 cells were cultured on coverslips for the IFA [25]. In brief, 10^6^ C6/36 cells were plated in six-well culture plates for 24 h. A DENV2 suspension was added to each well, allowed to be adsorbed for 1 h, and then incubated for more time as needed. Cells were fixed with 4% paraformaldehyde for 30 min and subsequently treated with 0.1% Triton X-100 for 2 min to increase the permeability. Primary antibodies included a rabbit anti-BiP/GRP78 antibody (homemade products) and monoclonal antibodies of the anti-envelope (E) protein of flaviviruses (from Dr. Oscar Guei-Chuen Perng, National Cheng Kung University, Taiwan), followed by secondary antibodies of DyLight 594-conjugated goat anti-rabbit immunoglobulin G (IgG, Thermo Scientific, Waltham, MA, USA) and Alexa Fluor 488-conjugated goat anti-mouse IgG (Life Technologies, Carlsbad, CA, USA), respectively, to detect specific proteins within cells. Finally, 4′-6-diamidino-2-phenylindole (DAPI), which presents a blue color, was used as an indicator of cell nuclei. Prepared specimens were observed under a laser scanning confocal microscope (Zeiss LSM 510, Vertrieb, Germany). One of three replicates is shown in the figures.

### 2.13. Construction of a MicroRNA- (miR-) Based RNA Interference (RNAi) Vector for Knockdown of the BiP/GRP78 Gene

This approach followed a protocol to generate a double-stranded oligo as a tool of miR RNAi (Invitrogen), as briefly described in a previous report [17]. The miR RNAi sequence to knock down the BiP/GRP78 gene (miR-BiP/GRP78) was generated by annealing top- and bottom-strand oligos containing linkers (top: 5′TGCTG…3′; bottom: 5′CCTG…C3′), the mature miR-BiP/GRP78 antisense target sequence, the loop sequence, and the sense target sequence (5′-AAGAATCATGACCACCAAT-3′), forming double-stranded- (ds-) oligos (top strand: 5′-TGCTGATTGGTGGTCAGCTGATTCTTGTTTTGGCCACTGACTGACAAGAATCATGACCACCAAT-3′ and bottom strand: 5′-CCTGATTGGTGGTCATGATTCTTGTCAGTCAGTGGCCAAAACAAGAATCAGCTGACCACCAATC-3′). The constructed ds-oligos were subsequently ligated to a miR cassette in the pcDNA™ 6.2-GW/EmGFP-miR vector. This miR cassette contained a murine miR-155 flanking sequence that enhances miR formation in mammal cells as well. In addition, there was a reporter gene, EmGFP, upstream of the miRNA cassette to facilitate the selection of successfully transfected cells. The insert containing EmGFP and miR-BiP/GRP78 was amplified by a PCR using primer pair EcoR1-mi-F (AAAGAATTCCTAGTTAAGCTATCAACAAGTTTG) and mi-R-Notl (TTTGCGGCCGCATCAACCACTTTGTACAAGAAAG) and inserted into the pAC5.1-miR-neo vectors, which were then transfected into C6/36 cells. Subsequently, transfected cells were selected by treatment with G418 (Sigma) and sorted four times by FACScan flow cytometry in sequence. Transcript changes of the BiP/GRP78 gene in cells, either transfected or not, were validated by a real-time RT-qPCR with forward (5′-CCTACTCGTGTGTGGGAGTGTA-3′) and reverse (5′-ATGATACGCATCACGTTCAAAC-3′) primers.

### 2.14. Identification of XBP1 Messenger RNA- (mRNA-) Splicing Activity

Based on a 706 bp fragment encoding a hypothetical protein of* Ae. aegypti* (accession number: AAEL005558-RA) highly similar to the* XBP1* gene of* Homo sapiens*, XBP1 cDNA of C6/36 cells was obtained and used as a template for sequencing and detecting mRNA splicing via a conventional RT-PCR with the primers Xbp1(s)-F (5′-ATCTCCAGCAGCACCTACA-3′) and Xbp1(s)-R (5′-TTGTGACATTAGCGAGGAG-3′). In order to obtain a better resolution of electrophoresis for the PCR products (with a predicted size of 308 bp), a 2% agarose gel was used in a run at 100 V for 40 min. The band pattern can be of two types, appearing as a single band (unspliced form) or as two bands (spliced form), which was used to identify the splicing activity of XBP1 mRNA. The deleted fragment was identified by comparing sequences from PCR products extracted from two specific bands on the gel. One of three replicates is shown in the figures.

### 2.15. Knockdown of XBP1 Prepared by Corresponding dsRNA

A 515 bp fragment of dsRNA to knock down XBP1 was prepared following the procedure used to prepare XBP1 dsRNA described above, except for the appropriate designed primers of dsXBP1-F (5′-TAATACGACTCACTATAGGGAGAGTCGCCGCACAAACTTCACGC-3′) and dsXBP1-R (5′-TAATACGACTCACTATAGGGAGAGTCCCGCATCAGCTCCTTCCAG-3′). For confirmation of the knockdown efficiency by a conventional RT-PCR, the primer pair used was dsXBP1(1)-F (5′-CACTGAGTCAGGACAGGACGACG-3′) and dsXBP1(1)-R (5′-GGA TTCCAGCTGTTCTGGTGGG-3′). One of three replicates is shown in the figures.

### 2.16. Coimmunoprecipitation (Co-IP) and Western Blotting

HAeGFPBiP/GRP78- or HAeGFP-expressing cells infected with DENV2 at an MOI of 1 for 24 h were lysed with CelLytic M Cell Lysis Reagent (Sigma-Aldrich) at 4°C for 20 min. After removing cellular debris (4°C, 12,000 ×g, 15 min), human influenza hemagglutinin (HA)BiP/GRP78 and HAeGFP proteins were immunoprecipitated using an Anti-HA Immunoprecipitation Kit (Sigma-Aldrich). Immunoprecipitated HABiP/GRP78, HAeGFP, and their associated proteins were analyzed by Western blotting to verify the identity of the protein [28]. For Western blotting, extracted proteins were electrophoresed on 12% (w/v) sodium dodecylsulfate polyacrylamide gel electrophoresis (SDS-PAGE) in nonreducing conditions [25]. Separated proteins were then transferred onto Immobilon™-P Transfer Membranes (Millipore, Billerica, MA, USA). After blocking with 5% milk-TBS-0.1% Tween 20 buffer at room temperature for 1 h, membranes were probed by the indicated primary and subsequently corresponding secondary antibodies at room temperature for 1 h. After a final washing, membranes were visualized with Immobilon Western Chemiluminescent HRP Substrate (Millipore), and signals were detected by Fuji X-ray film (FUJIFILM, Tokyo, Japan). The primary antibodies used for this experiment included specific anti-E protein monoclonal antibodies as well as rabbit antiserum against BiP/GRP78 and eGFP/HA tags, while the secondary antibody was goat anti-mouse (or rabbit) IgG conjugated with peroxidase (Chemicon, Temecula, CA, USA). One of three replicates is shown in the figures.

### 2.17. Statistical Analysis

Comparisons between two means were analyzed by Student's *t*-test, while three or more means from a given time period were analyzed by a one-way analysis of variance (ANOVA) at a 5% level of significance.

## 3. Results

### 3.1. Induction of the Oxidative Stress by DENV2 Infection in C6/36 Cells

**Figure 1:**
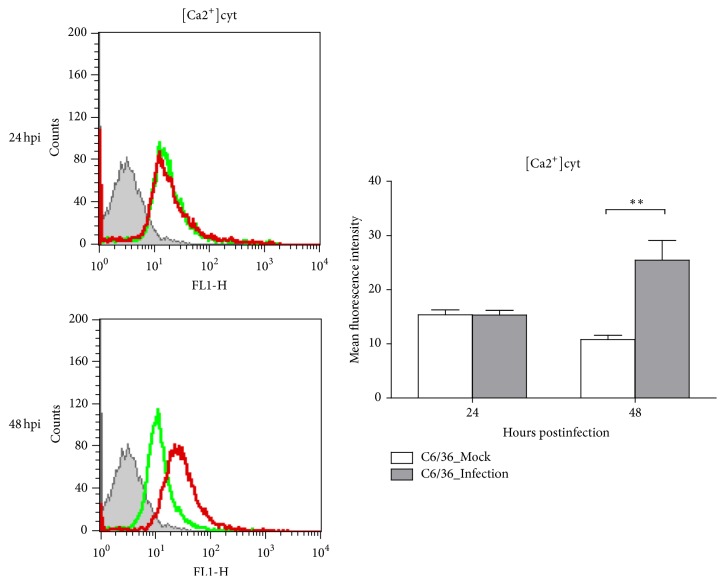
Oxidative stress induction in dengue 2 virus- (DENV2-) infected C6/36 cells. Based on flow cytometry, the cytosolic free calcium concentration ([Ca^2+^]cyt) in DENV2-infected-C6/36 cells had slightly changed at 24 h postinfection (hpi), while it had obviously risen at 48 hpi. This reflects that endoplasmic reticular (ER) stress was activated by DENV2, even though most infected cells remained undamaged throughout the period of observation. Quantitatively, the difference in concentrations, as measured by the fluorescence intensity, between infected and uninfected cells remained low at 24 hpi but had significantly increased by 48 hpi (*p* < 0.05; Student's *t*-test). Green line: uninfected C6/36 cells; red line: DENV2-infected C6/36 cells.

In C6/36 cells infected with DENV2 at an MOI of 1, the spatiotemporal distribution of intracellular calcium had slightly changed at 24 hpi but had obviously increased at 48 hpi. The concentration of cytosol free calcium ([Ca^2+^]cyt), quantified by measuring the fluorescence intensity, revealed no change in C6/36 cells infected by the DENV2 until the cells had been infected for at least for 48 h (*p* < 0.01; Student's *t*-test) (Figure 1). The results presented here indicated that DENV2-induced oxidative stress leads to accumulation of ROS in infected C6/36 cells, which is compatible with our previous observations [17].

### 3.2. Upregulation of BiP/GRP78 in DENV2-Infected C6/36 Cells

**Figure 2:**
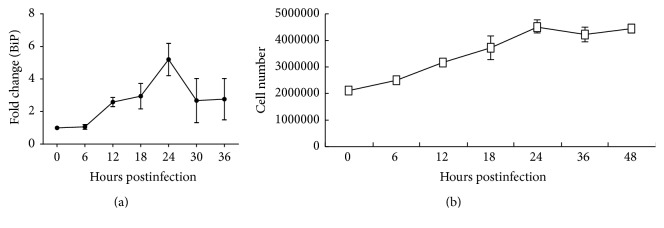
Upregulation of BiP/GRP78 in C6/36 cells with dengue 2 virus (DENV2) infection. (a) The expression of BiP/GRP78 mRNA in C6/36 cells infected with DENV2 was validated by a real-time RT-qPCR, showing that the expression of this gene significantly changed along with the time of infection (*p* < 0.05; one-way ANOVA). Specifically, it remained unchanged (1.06-fold) at 0 and 6 h postinfection (hpi) but had increased 2.58-fold by 12 hpi and 2.94-fold by 18 hpi and had reached a peak (5.20-fold) by 24 hpi. BiP/GRP78 mRNA levels had subsequently fallen back to 2.67- and 2.76-fold increases at 30 and 36 hpi, respectively. (b) The number of cells infected with DENV2 increased about 2-fold between the initial inoculation (at 0 hpi) and 24 hpi, and a stable level then remained from 36 to 48 hpi. This reveals that the change in BiP/GRP78 was not the effect of cell growth.

Based on the results of a real-time RT-qPCR, the BiP/GRP78 expression level significantly changed over time with DENV2 infection in C6/36 cells (*p* < 0.05; one-way ANOVA). Its expression level at 6 hpi (1.06-fold) remained around the baseline compared to that measured in mock-infected cells. The expression level subsequently rose 2.58- and 2.94-fold at 12 and 18 hpi, respectively, and had reached a peak (5.20-fold) at 24 hpi. Afterward, the increasing trend of the BiP/GRP78 mRNA level reversed 2.67- and 2.76-fold at 30 and 36 hpi, respectively (Figure 2(a)). The number of DENV2-infected C6/36 cells increased about 2-fold between the initial inoculation (at 0 hpi) and 24 hpi, followed by a stable level at 36~48 hpi (Figure 2(b)), revealing that the change in BiP/GRP78 was not the result of cell growth but was an indicator of stress in infected cells.

### 3.3. XBP1 Expression and Splicing Activity in Response to DENV2 Infection in C6/36 Cells

**Figure 3:**
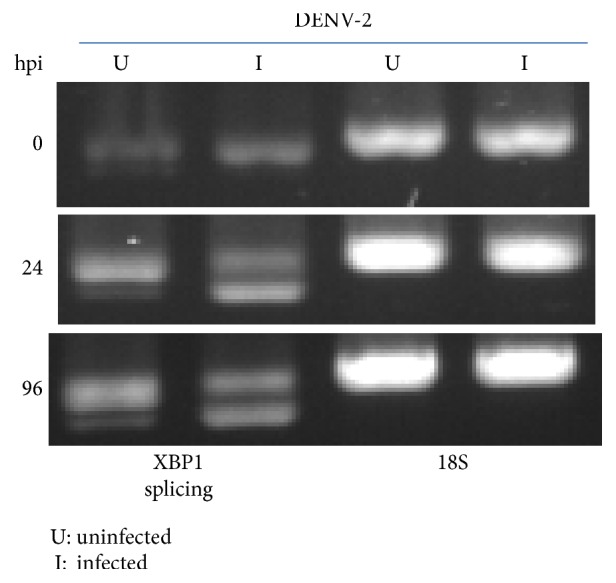
Dengue 2 virus (DENV2) activates XBP1 expression and its splicing activity in C6/36 cells. In C6/36 cells without infection by DENV2, most XBP1 mRNA was intact (unspliced), while its spliced form was seen at 24 h postinfection (hpi) and had risen to a higher level by 96 hpi. This indicates that the endoplasmic reticular (ER) stress induced by DENV2 in mosquito cells also triggers, in addition to BiP/GRP78 expression, XBP1 mRNA expression and its splicing activity.

In order to measure the change in XBP1 mRNA in response to DENV2 infection in C6/36 cells, a 308 bp fragment derived from a hypothetical protein of* Ae. aegypti* was selected for amplification (Figure S1 in Supplementary Material available online at https://doi.org/10.1155/2017/3519158). There were usually two bands including the original sequence of XBP1 mRNA (uXBP1) and a shorter sequence or spliced form (sXBP1) with 23 nt deleted (5′-TCCGCCCAAACCCCTCAGCAGCA-3′) in DENV2-infected C6/36 cells. In general, only uXBP1 was detected in mock-infected cells even though they had been cultured for 96 h. Nevertheless, sXBP1 had appeared by 24 hpi and was at an even higher level at 96 hpi (Figure 3). This indicates that splicing activity of XBP1 mRNA possibly occurs in response to the ER stress induced by DENV2 infection in C6/36 cells, producing a form which functions as a transcription factor of downstream genes [29]. To learn more about the gene sequence of XBP1 from C6/36 cells, a 660 nt sequence (without the 44 nt of primers derived from the sequence of* Ae. aegypti* and the last 2 nt in the original amplified sequence) representing a partial sequence of BiP/GRP78 was sequenced and submitted to NCBI (accession number: KU672624) as shown in Figure S2.

### 3.4. Reduction of BiP/GRP78 in XBP1-Knockdown C6/36 Cells with DENV2 Infection

**Figure 4:**
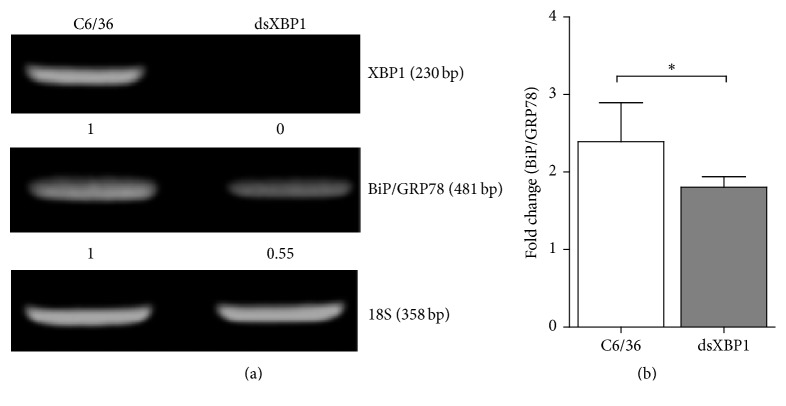
Involvement of XBP1 in regulating BiP/GRP78 transcription in C6/36 cells with dengue 2 virus (DENV2) infection. (a) A 515 bp fragment chosen from a known sequence of XBP1 mRNA was used as the target to form a double-stranded RNA (dsRNA) segment, which was then applied to knock down the XBP1 mRNA in C6/36 cells. The results from a conventional RT-PCR showed that XBP1 mRNA was efficiently knocked down in cells transfected with dsRNA of XBP1 (relative density was close to 0 for the specific band compared to that in cells without transfecting dsRNA of XBP1). Reduced BiP/GRP78 expression was also detected in DENV2-infected cells with knockdown of XBP1 compared to control cells without treatment (relative density was shown about 0.55 compared to that in untreated cells). (b) By using a real-time PCR (RT-PCR) to quantitate the expression level of BiP/GRP78 in C6/36 cells with DENV2 infection for 24 h, its increase in multiples of change was significantly lower in C6/36 cells with knockdown of XBP1 (*p* < 0.05; Student's *t-*test). This indicates that XBP1 may play a role in upregulating BiP/GRP78 expression in C6/36 cells after infection with DENV2.

A 515 bp fragment chosen from the sequence of XBP1 mRNA mentioned above was used as the target for designing a dsRNA segment, which was then applied to knock down the expression of XBP1 mRNA in C6/36 cells. The results showed that XBP1 mRNA including spliced and unspliced forms was efficiently reduced as shown in Figure 4(a), in which BiP/GRP78 was also expressed at a lower level compared to that of the control group according the result from a conventional RT-PCR. Using an RT-qPCR for quantification, BiP/GRP78 expression was only 1.80-fold in DENV2-infected cells (24 hpi) with XBP1 knockdown, while it was 2.39-fold in the control group, indicating a significant difference between the two groups in the expression responsible for DENV2 infection (Student's *t*-test; *p* < 0.05) (Figure 4(b)). The results suggested that BiP/GRP78 expression in DENV2-infected mosquito cells is highly associated with the amount of XBP1 mRNA, supposedly the spliced form. This suggested that BiP/GRP78 could be one of the downstream genes using XBP1 as a transcription factor.

### 3.5. Reduction of DENV2-Induced Endoplasmic Reticular (ER) Stress by the Overexpression of BiP/GRP78

In order to investigate the effect of BiP/GRP78 on regulating the induction of ER stress, accumulation of superoxide anions was measured in BiP/GRP78-overexpressing C6/36 cells. To prepare the BiP/GRP-overexpressing vector, the full-length sequence of the BiP/GRP78 ORF containing 1971 nucleotides (nt) was obtained based on a newly identified partial sequence. The sequence was submitted to NCBI (accession number: KU672623) and is shown in Figure S3. Alignment of the full-length BiP/GRP78 ORF sequences among mosquitoes showed that BiP/GRP78 derived from C6/36 cells was 91% (176 nt difference) and 89% (227 nt difference) similar to those of* Ae. aegypti* (accession number: DQ440225) and* Culex quinquefasciatus* (accession number: XM_00814566), respectively. In turn, we determined 656 amino acids (aa) from C6/36 cells, which possessed one extra amino acid compared to those derived from* Ae. aegypti* (99% similarity), or a 27 aa difference with that of* Cx*.* quinquefasciatus* (96% similarity) which contains 657 aa (Figure S4).

**Figure 5:**
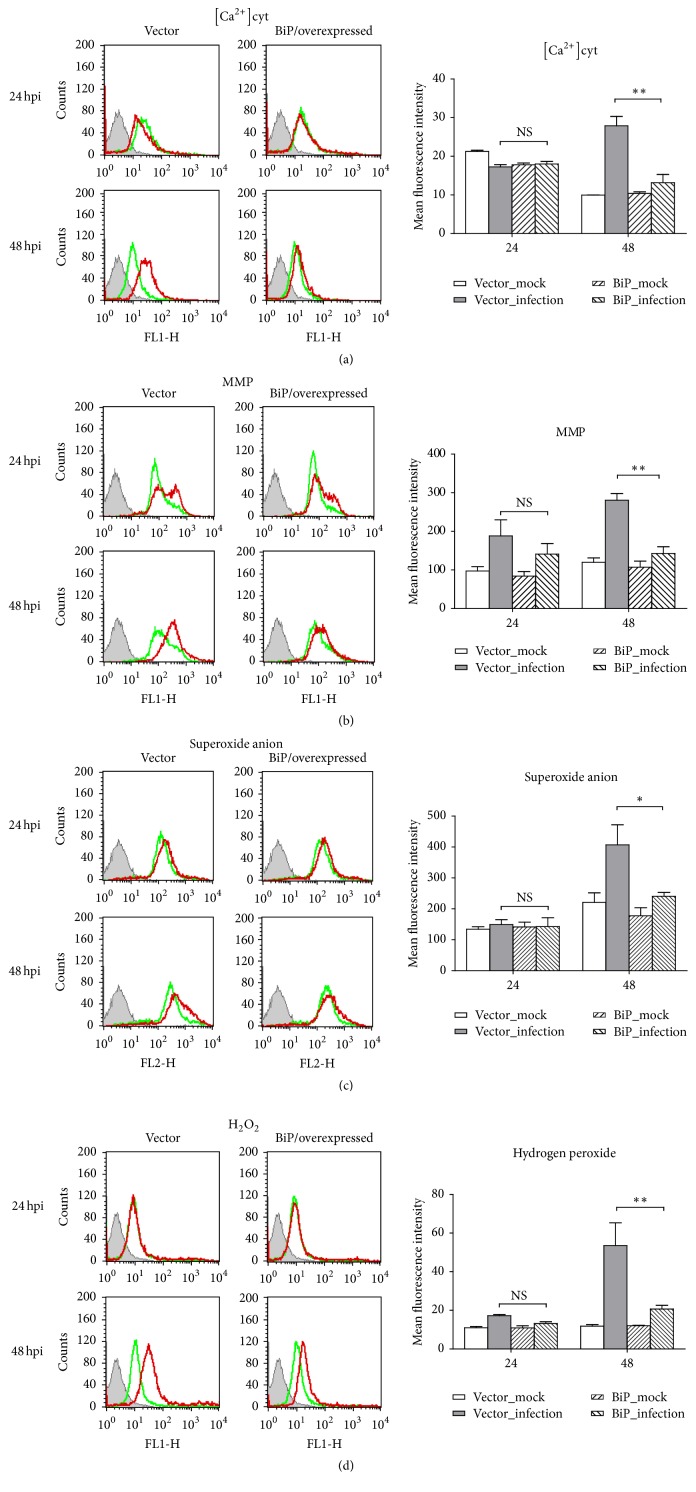
Alleviation of oxidative stress by BiP/GRP78 overexpressed in C6/36 cells with dengue 2 virus (DENV2) infection. (a) In DENV2-infected C6/36 cells transfected with the BiP/GRP78-overexpressing vector, the cytosolic free calcium ([Ca^2+^]cyt) concentration induced by DENV2 infection was not significantly reduced until 48 hpi (Student's *t*-test; *p* < 0.01). (b) Significant alleviation of the change in the mitochondrial membrane potential (MMP) was shown in DENV2-infected C6/36 cells at 24 and 48 h postinfection (hpi) (Student's *t*-test; *p* < 0.01). (c) Superoxide anions that accumulated in infected C6/36 cells with BiP/GRP78 overexpression were reduced, especially at 48 hpi (Student's *t*-test; *p* < 0.05). (d) There was a similar changing trend to the situation with superoxide anions for the accumulation of H_2_O_2_ which was also alleviated in BiP/GRP78-overexpressing C6/36 cells (Student's *t*-test; *p* < 0.01). Green line: uninfected C6/36 cells; red line: DENV2-infected C6/36 cells.

In DENV2-infected C6/36 cells transfected with a BiP/GRP78-overexpressing vector, the concentration of [Ca^2+^]cyt was significantly reduced at 48 hpi (Student's *t*-test; *p* < 0.01) as [Ca^2+^]cyt only showed an increase at this time point (Figure 5(a)). However, significant alleviation of the MMP was shown in cells at 48 hpi (Student's* t*-test; *p* < 0.01) though only a slight different was shown at 24 hpi (Figure 5(b)). Furthermore, superoxide anions (Figure 5(c)) and H_2_O_2_ (Figure 5(d)) also found to have accumulated in infected C6/36 cells with overexpression of BiP/GRP78 were alleviated, especially at 48 hpi (Student's *t*-test; *p* < 0.05 and 0.01, resp.). Results indicated that oxidative stress can be induced by DENV2 due to an altered intracellular distribution of calcium ions, followed by altered MMPs. According to the present results, both of these can be alleviated by the overexpression of BiP/GRP78 which was shown to be upregulated in infected C6/36 cells, leading to recovery from the ER stress induced by DENV2.

### 3.6. Involvement of BiP/GRP78 in DENV2 E Protein Synthesis

**Figure 6:**
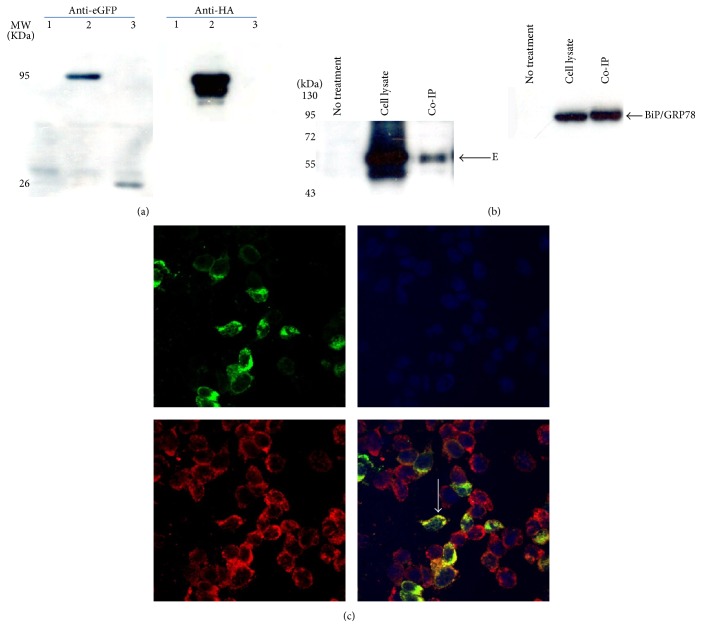
Association of BiP/GRP78 with synthesis of the dengue 2 virus (DENV2) envelope (E) protein in C6/36 cells. For the Co-IP assay, both an enhanced green fluorescent protein (eGFP) and human influenza hemagglutinin- (HA-) tagged BiP/GRP78-overexpressing vector were constructed and transfected into C6/36 cells. (a) The successful expression of BiP/GRP78 was validated with eGFP and HA antibodies in Western blot analysis. Nothing was seen in lane 1 which represents C6/36 cells neither overexpressing BiP/GRP78 nor infected with DENV2. BiP/GRP78 can be detected in the same position of the band by both antibodies in lane 2 which represents DENV2-infected C6/36 cells transfected with the BiP/GRP78-overexpressing vector tagged with eGFP and HA. Only eGFP can be detected as shown in lane 3 which represents C6/36 cells transfected with a vector only expressing eGFP. (b) Co-IP results revealed that overexpressed BiP/GRP78 in C6/36 cells eventually interacted with the viral E protein that was also detected in the lysate of cells infected with DENV2 for 24 h. Meanwhile, BiP/GRP78 was also identified in the same cells. Lane 1:  C6/36 cells that had neither BiP/GRP78 overexpression nor DENV2 infection (no treatment). Lane 2: DENV2-infected C6/36 cells transfected with BiP/GRP78 (cell lysate). Lane 3: IP results from DENV2-infected C6/36 cells transfected with BiP/GRP78 and infected with the DENV2 (Co-IP). (c) Confocal microscopy demonstrated that BiP/GRP78 was upregulated in C6/36 cells with DENV2 infection at 24 h postinfection (hpi) and colocalized with the viral envelope (E) protein, as shown in the merged image. Green: E protein of DENV; blue: DAPI; red: BiP/GRP78.

After transfection of a BiP/GRP78-overexpressing vector tagged with both HA and eGFP into DENV2-infected C6/36 cells, BiP/GRP78 was readily identified by both anti-HA and anti-eGFP antibodies through a Western blot analysis (Figure 6(a)). Co-IP results from transfected cells with DENV2 infection clearly revealed that BiP/GRP78 binds to the viral E protein (Figure 6(b)). The results reflected that BiP/GRP78 may be involved in the synthesis of the E protein of DENV2 in mosquito cells. Meanwhile, BiP/GRP78 was revealed to colocalize with viral E protein in C6/36 cells infected with DENV2 for 24 h according to observations with confocal microscopy (Figure 6(c)).

### 3.7. Effect of BiP/GRP78 on the Synthesis of the Viral E Protein in C6/36 Cells

**Figure 7:**
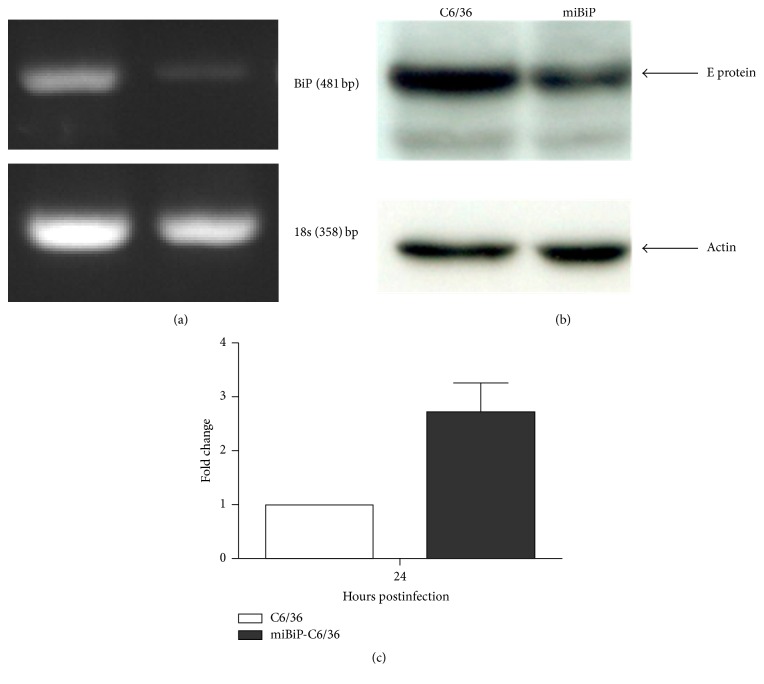
Effect of BiP/GRP78 on the synthesis of the viral envelope (E) protein in C6/36 cells. (a) A microRNA-based BiP/GRP78 knockdown system (miBiP) was constructed; the efficient reduction of BiP/GRP78 expressed by C6/36 cells was seen. (b) Viral RNA expression had increased up to 2.5-fold by 24 hpi in C6/36 cells with BiP/GRP78 knockdown. (c) A lower level of viral E protein was detected in dengue 2 virus- (DENV2-) infected C6/36 cells (24 h postinfection (hpi)) with BiP/GRP78 knockdown compared to cells without knockdown. Results indicated that BiP/GRP78 was associated with viral E protein synthesis but had no effect on viral RNA replication.

To demonstrate the effect of BiP/GRP78 on the DENV2 viral E protein in C6/36 cells, an efficient miR-based knockdown system was established (Figure 7(a)). In these cells, synthesis of the viral E protein decreased to a level lower (67.5%) than in cells without BiP/GRP78 knockdown after normalization to actin amounts in corresponding groups (Figure 7(b)). However, the expression of viral RNA had significantly increased ~2.5-fold at 24 hpi in DENV2-infected C6/36 cells in which BiP/GRP78 was knocked down (Student's *t*-test; *p* < 0.05) (Figure 7(c)). It reveals there was no effect of BiP/GRP78 on viral RNA replication in DENV-infected C6/36 cells. This indicated that BiP/GRP78 possessed effects only on protein synthesis and ultimately not on RNA replication. Plaque assay results showed that the virus titer in the knockdown group (3.24 × 10^5^ PFU/ml) was reduced to 64.4% compared to that of the control group (5.03 × 10^5^ PFU/ml). It further suggested that the effect of BiP/GRP78 occurs in the synthesis of the viral E protein and subsequent formation of viral particles.

## 4. Discussion

DENV infection in human or other mammal cells mostly leads to apoptosis due to persistent oxidative stress during the UPR [30]. Being a virus transmitted by mosquito vectors, DENVs can also infect cells of specific mosquitoes. We previously demonstrated that DENVs can also infect mosquito cells, causing ER stress which is usually alleviated by antioxidant defense and antiapoptotic effects [17, 18]. Herein, we detected an obvious change in the spatiotemporal distribution of intracellular calcium (Ca^2+^) within those cells: usually [Ca^2+^]cyt was shown to have increased at 48 hpi. This indicates that intracellular Ca^2+^ signaling may have been impinged by this time point [31], since [Ca^2+^]cyt normally serves as an intracellular signaler [32–34]. In addition, we repeatedly demonstrated that the MMP is altered in C6/36 cells with DENV2 infection, compatible with our previous observations [17]. This suggests that a proapoptotic potential was induced, as the MMP may reflect a rate-limiting event of apoptosis induced by viral proteins [35]. Specifically, alteration of the MMP was detected at as early as 24 hpi, much earlier than the situation with [Ca^2+^]cyt. Presumably, transfer of Ca^2+^ stored in the ER to mitochondria was earlier than that to the cytosol [36, 37]. This may further confirm the theory which proposes that mitochondria could be Ca^2+^ buffers [38].

Superoxide anions are a component of ROS that serve as an upstream modulator of stimuli-induced H_2_O_2_ in cells [39]. In this study, both superoxide anions and H_2_O_2_ were found to be induced and accumulated in infected cells, more evidently at 48 hpi. This indicates that DENV2 infection in mosquito cells is able to induce oxidative stress via a cascade involving ER Ca^2+^ depletion, MMP changes, and ROS accumulation [9]. Although oxidative stress is induced by DENVs in mammalian cells, it can also activate antioxidant defenses and antiapoptotic effects in mosquito cells [17, 18]. Our previous results showed that it may help mosquito cells achieve a higher possibility of surviving DENV2 infection [15]. Eventually, accumulation of these ROS is mostly alleviated via overexpression of recombinant BiP/GRP78 mostly through assisting viral protein folding during the UPR.

BiP/GRP78 is an established marker for ER stress due to its ability to activate transmembrane ER stress sensors (IRE1, PERK, and ATF6), inducing three signaling pathways during the UPR [40]. It is known that BiP/GRP78 is a residential protein that is present at a lower level in the ER in the absence of stimuli or stress [41]. However, it is upregulated in response to viral infections including DENV in mammalian cells [42] and serves as a chaperone involved in the event of protein folding [43]. It may direct cells back to normal or to apoptosis in response to ER stress induced by the UPR [44]. In the present study, significant upregulation of BiP/GRP78 was observed in parallel with the induction of oxidative stress in DENV2-infected C6/36 cells.

Of the three signaling pathways activated during the UPR, ATF6 may function to induce the production of X-box binding protein 1 (uXBP1) in response to ER stress [45]. It is followed by splicing activity to create spliced forms of XBP1 (sXBP1) via a process of a uXBP1 reading frame shift mediated by IRE1 signaling [29, 46]. The present study shows that uXBP1 is increasingly produced along with the expression of BiP/GRP78 over time with DENV2 infection in mosquito cells. XBP1 splicing was evidenced by a deletion of a 23 nt sequence from an identified fragment of uXBP1, leading to the formation of sXBP1 in C6/36 cells with DENV2 infection. In fact, XBP1 splicing activity was reported in mammalian cells infected with flaviviruses such as West Nile virus, Japanese encephalitis virus, and DENV [47, 48]. This reveals that sXBP1 plays an important role, presumably as a transcription factor critical to the expression of various chaperones [46]. When we used the dsRNA technique to knock down the total amount of XBP1, BiP/GRP78 expression was significantly reduced. This suggests that upregulation of BiP/GRP78 in DENV2-infected C6/36 cells may be modulated by XBP1 expression and subsequent splicing activity in response to DENV2 infection. BiP/GRP78 is generally located in the ER lumen and functions to bind newly synthesized proteins to maintain them in a competent state for appropriate folding and oligomerization [12, 49], leading to reduced oxidative stress induced by DENV infection [48].

BiP/GRP78 can be observed to colocalize with the viral E protein by confocal microscopy. Their potential interactions were further demonstrated via a Co-IP assay in C6/36 cells infected with DENV2. The synthesized E protein of DENV2 obviously decreasing in cells with BiP/GRP78 knockdown suggests that this DENV2-induced chaperone is critically important for the synthesis of viral proteins in mosquito cells. Since the efficiency of viral RNA replication was not suppressed by knockdown of BiP/GRP78, this chaperon in mosquito cells might only possess effects related to viral protein synthesis but not its RNA replication. It was reported that BiP/GRP78 plays a role in the quality control of synthesized viral proteins to ensure that only correctly folded proteins are translocated and used for virus assembly [50]. This feature, in turn, would elevate survival opportunities for mosquito cells with DENV2 infection via maintenance of ER homeostasis, which may be disrupted by viral infections [51]. In fact, obvious accumulation of ROS (superoxide anions and hydrogen peroxide) was observed in DENV2-infected C6/36 cells in the early stage of infection, and it was consequently ameliorated by upregulation of BiP/GRP78. Results from the overexpression of recombinant BiP/GRP78 actually prove this possibility. BiP/GRP78 upregulated by DENV2 infection in mosquito cells could be an essential factor to maintain ER homeostasis that is required to increase the survival rate of infected cells.

Taken together, synthesized DENV2 proteins accumulating in the ER initiated the UPR that markedly induced oxidative stress in mosquito cells, resulting in elevated production of uXBP1 and subsequent splicing activity as shown in mammalian cells at the earlier stage of infection. sXBP1 then upregulated BiP/GRP78 to further assist with correct protein folding, resulting in reduced virus-induced ER stress and a higher survival rate of infected mosquito cells. It may improve our knowledge of virus/vector interactions for viral replication in mosquito cells. This interesting finding may also elucidate how mosquito vectors can remain healthy to transmit DENVs efficiently in nature.

## Supplementary Material

The supplementary materials include partial sequences of XBP1 derived from C6/36 cells; in which the primer pair used to detect its splicing activity and the fragment (23 nucleotides) expected to be deleted in response to the stress are included. In addition, the nucleotides and deduced amino acids of the BiP/GRP78 open reading frame derived from C6/36 cells were also shown in this part.
