# Supplementary material for: XBP1-Mediated BiP/GRP78 Upregulation Copes with Oxidative Stress in Mosquito Cells during Dengue 2 Virus Infection
Source: Biomed Res Int. 2017 Oct 1;2017:3519158. doi: 10.1155/2017/3519158 (PMC5642879; doi:10.1155/2017/3519158)

**Supplementary Figures and Legends**

The supplementary materials include partial sequences of XBP1 derived from C6/36 cells; in which the primer pair used to detect its splicing activity and the fragment (23 nucleotides) expected to be deleted in response to the stress are included. In addition, the nucleotides and deduced amino acids of the BiP/GRP78 open reading frame derived from C6/36 cells were also shown in this part.

**Fig. S1.** Partial sequence of XBP1 (308 nucleotides) derived from a hypothetical protein of *Aedes aegypti* was selected for amplification. This sequence included a short fragment (23 nucleotides) that was expected to be deleted in response to dengue 2 virus (DENV2) infection. The primers shown in the sequence are underlined, and those to be deleted are highlighted with bold and *italicized*.

**Fig. S2.** XBP1 is usually spliced in response to endoplasmic reticular (ER) stress. The spliced form (sXBP1) usually serves as a key component during the unfolded protein response (UPR). A sequence composed of 660 nucleotides (nt) without 44 nt of primers derived from the sequence of *Aedes aegypti* or the last 2 nt in the original amplified sequence was submitted to NCBI (accession no.: KU672624). The bolded fragment (in red) was deleted when splicing activity occurred.

**Fig. S3.** The full-length sequence of the BiP/GRP78 open reading frame (ORF) contained 1971 nucleotides (nt); which was submitted to NCBI (accession no.: KU672623). Alignment of full-length BiP/GRP78 ORF sequences among three mosquito species showed that BiP/GRP78 from C6/36 cells (derived from *Aedes albopictus*) was 91% (176 nt) and 89% (227 nt) similar to those from *Ae. aegypti* (accession no.: DQ440225) and *Culex quinquefasciatus* (accession no.: XM_00814566), respectively.

**Fig. S4.** Deduced 656 amino acids of the BiP/GRP78 open reading frame (ORF) from C6/36 cells (derived from *Aedes albopictus*) possessed 1 extra amino acid compared to that derived from *Ae. aegypti* with 99% similarity, and revealed a difference of 6 amino acids, including one missing in *Ae. aegypti*. In the meantime, the sequence from C6/36 cells lacked 1 amino acid possessed by *Culex quinquefasciatus*, leading to a difference of 27 amino acids or a 96% similarity.

Supplementary Figure S1


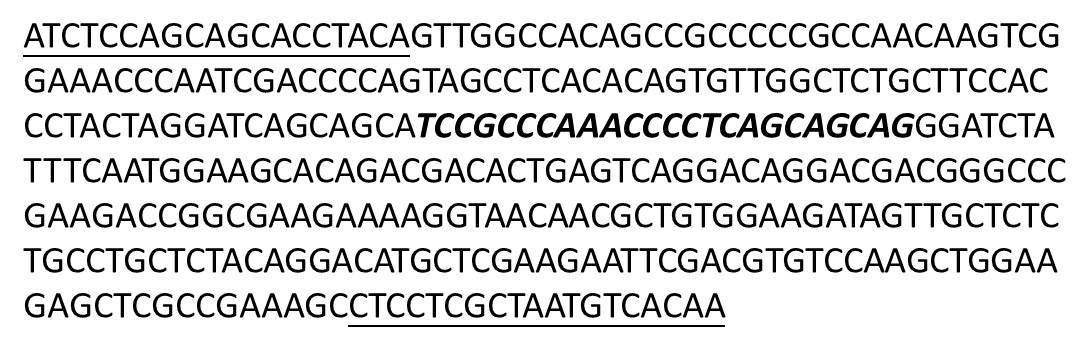


Supplementary Figure S2


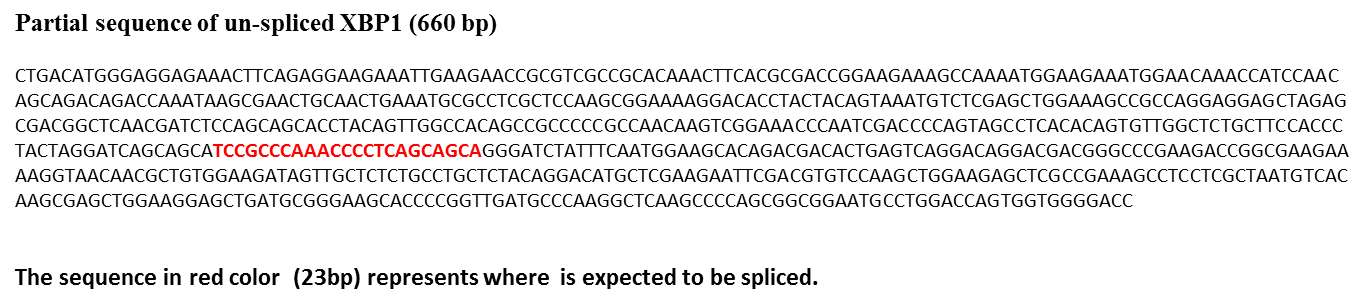


Supplementary Figure S3-1


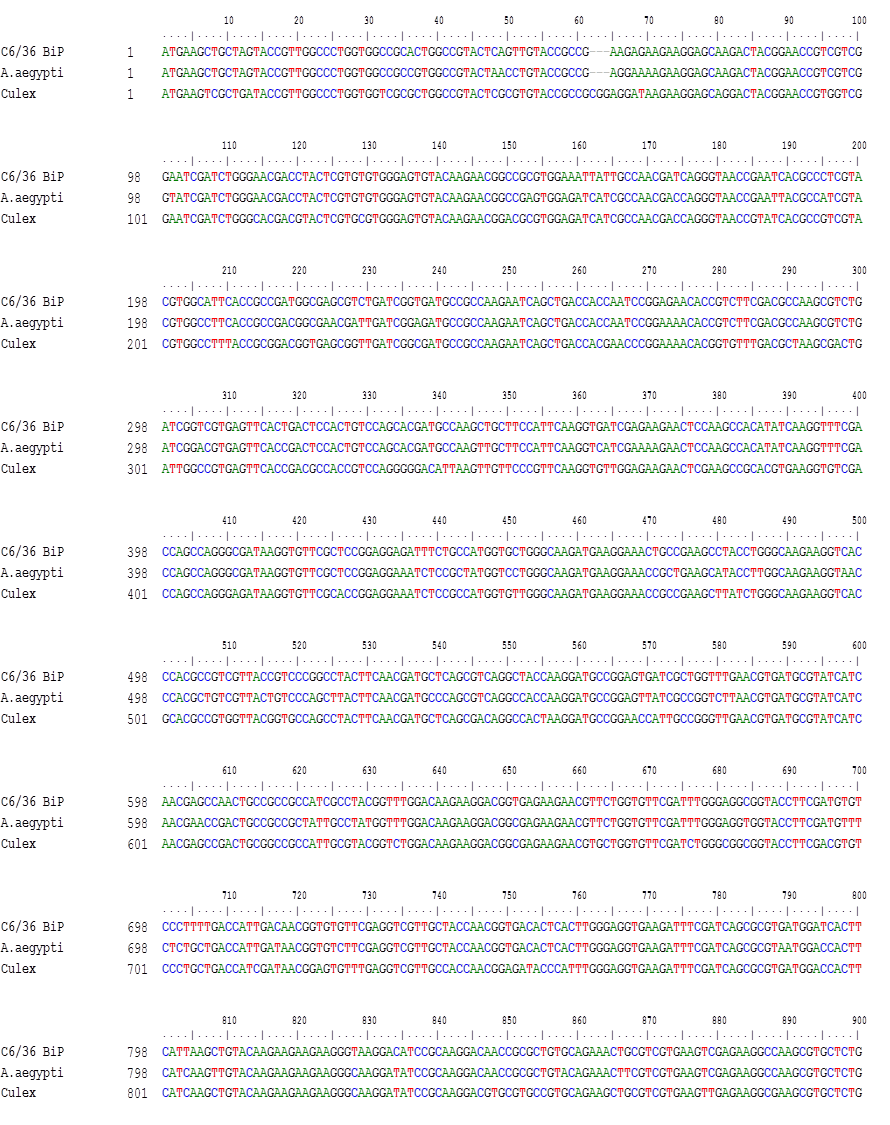


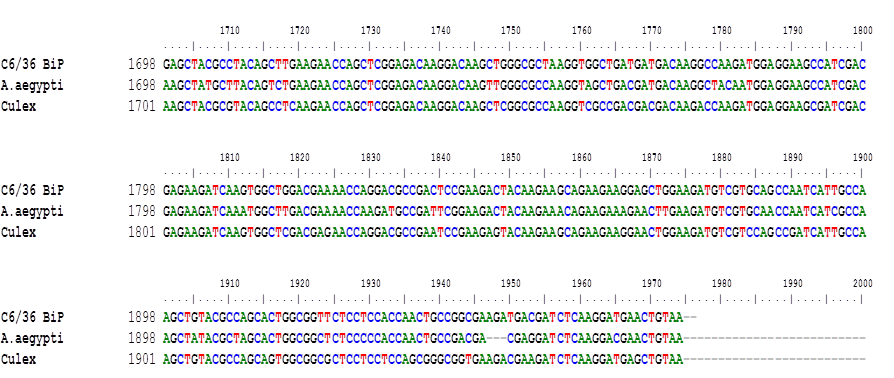


Supplementary Figure S3-2


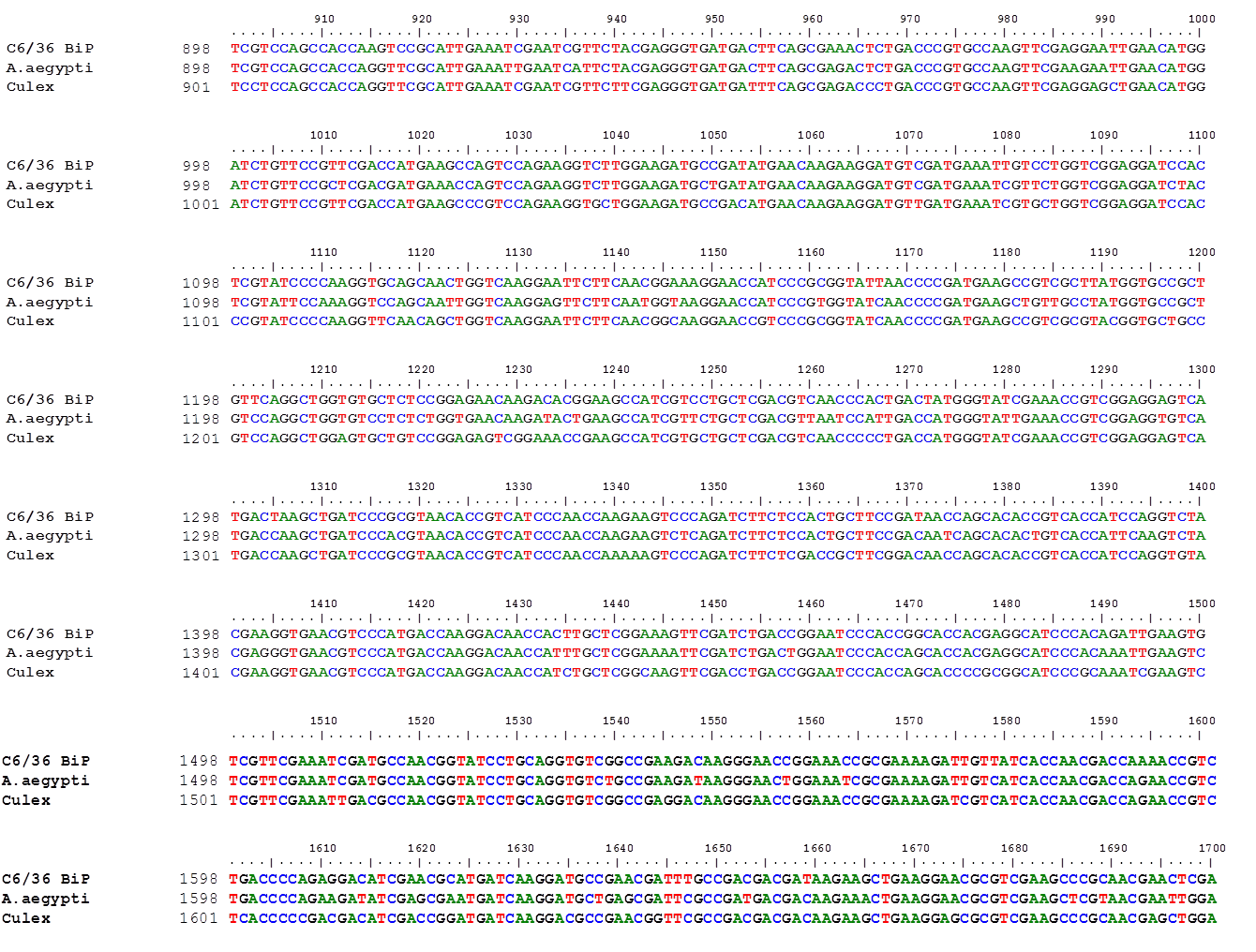


Supplementary Figure S3-3


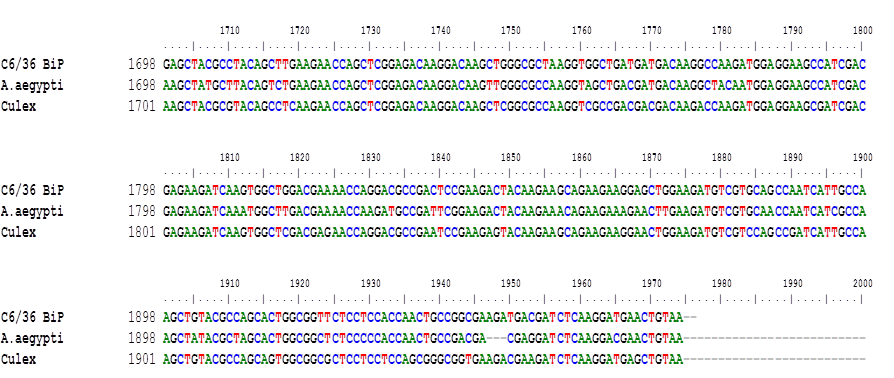


Supplementary Figure S4


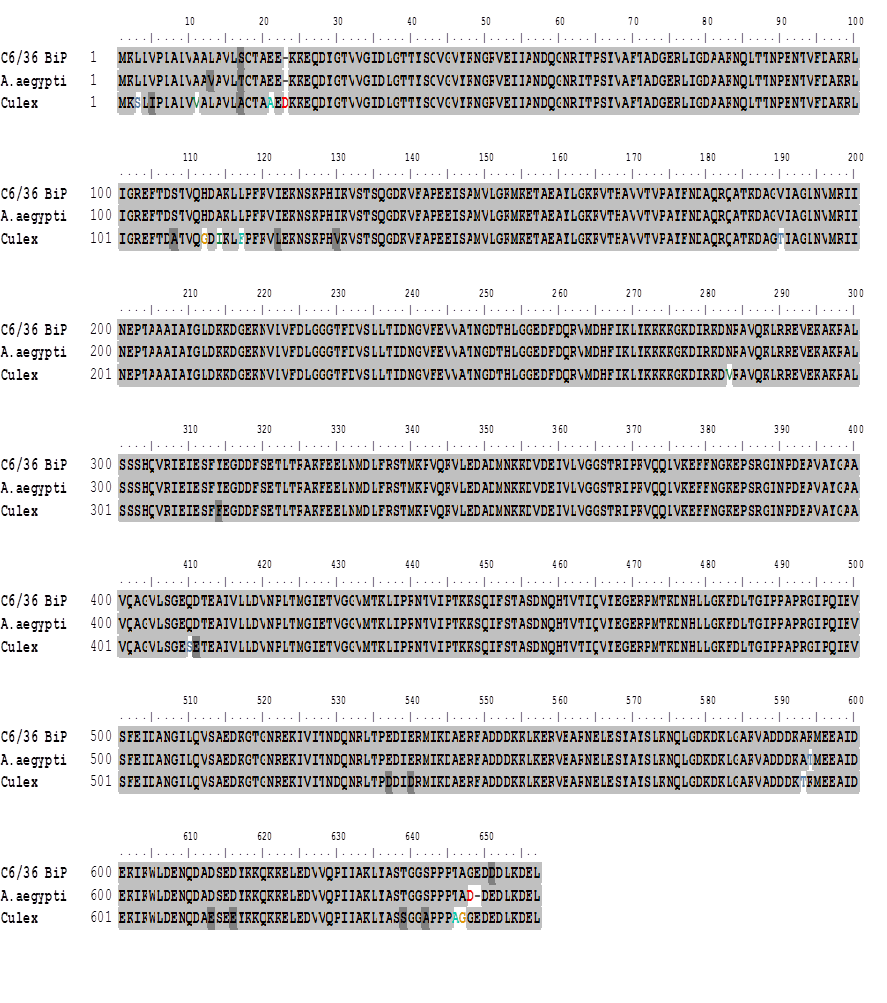

Supplement: Supplementary file 1 — The supplementary materials include partial sequences of XBP1 derived from C6/36 cells; in which the primer pair used to detect its splicing activity and the fragment (23 nucleotides) expected to be deleted in response to the stress are included. In addition, the nucleotides and deduced amino acids of the BiP/GRP78 open reading frame derived from C6/36 cells were also shown in this part. [file 3519158.f1.docx]
